# Assessing the Cross-Cultural Adaptation and Translation of a Text-Based Mobile Smoking Cessation Program in Samoa (TXTTaofiTapaa): Pilot Study

**DOI:** 10.2196/mhealth.9033

**Published:** 2018-08-31

**Authors:** Judith McCool, Helen Tanielu, Elaine Umali, Robyn Whittaker

**Affiliations:** ^1^ Department of Epidemiology and Biostatistics School of Population Health University of Auckland Auckland New Zealand; ^2^ Department of Sociology National University of Samoa Apia Samoa; ^3^ National Institute for Health Innovation School of Population Health University of Auckland Auckland New Zealand; ^4^ Institute for Health Innovation and Improvement (i3) Waitemata District Health Board Auckland New Zealand

**Keywords:** mHealth, mobile phone, Pacific, Samoa, tobacco cessation, text messages

## Abstract

**Background:**

Samoa faces a persistently high prevalence of adult tobacco use and few existing cessation support services. Mobile phones are ubiquitous and generally affordable.

**Objective:**

This study aimed to adopt a text message (short message service, SMS) smoking cessation program designed in New Zealand (stop smoking with mobile phones, STOMP) for use in Samoa to assist national objectives in reducing the tobacco use.

**Methods:**

Using focus groups with smokers and ex-smokers, we explored the context for tobacco use and preferences for SMS text messages. Postintervention focus groups were held after participants received SMS text messages for 1 week. Frequent face-to-face meetings with the primary partner (Ministry of Health Samoa) and key stakeholders contributed to the adaptation process. Participatory feedback and collaboration from stakeholders became an integral part of the cultural adaptation and translation of the program. Furthermore, detailed document analyses were included as part of the formal evaluation of the initiative to explore the core determinants of success in adapting the program to the Samoan cultural context.

**Results:**

The SMS text messages evolved remarkably following an iterative process of consultation, in situ testing, revision, and retesting to arrive at an acceptable country-specific version of the mobile smoking cessation program. The SMS text messages retained in the final set were consistent with the theory of behavioral change but reflected both linguistic and cultural nuances appropriate for Samoa. Adapting messages required simultaneous multilevel processes, including complex high-level engagement, between the team and the stakeholders, along with crafting the precise content for (character limited) messages.

**Conclusions:**

Receiving cessation support messages through a mobile phone is promising and appears to be an acceptable and accessible mode of delivery for tobacco cessation, particularly in the absence of alternative support. Adapting a text-based program in Samoa requires fastidious attention to the nuances of culture, language, and sociopolitical structures in the country.

## Introduction

Mobile phones can deliver programs to support behavioral change [1,2], data management or surveillance [3,4], and other essential public health activities. The use of mobile phones could potentially promote health and gender equity [5,6] and enhance community empowerment [7,8]. Short message service (SMS) text messaging, in particular, has been shown to be a simple and effective intervention for improving health service delivery in pregnant women [9,10], infectious disease cases [11,12], weight loss cases [13], and diabetes cases [14] and increasingly for smoking cessation support [15-18]. SMS text messages can present timely cues, behavioral reinforcement, and social support [19] all through an inexpensive delivery medium.

The increasing use of mobile phones in health care delivery, combined with its accessibility and relative affordability, underpins the growth of mobile-based programs in low- and middle-income countries (LMICs). The advantages of using mobile phones are especially emphasized when access to health services is limited to the lack of resources [20,21]. Innovative solutions requiring minimal resource and promising great impact are an attractive option driving the burgeoning number of mobile phone-based pilot studies in LMICs [22]. At present, there is a plethora of programs and lessons learned from pilot programs that many countries can leverage from to understand what works to minimize risks, provide savings, and increase impact [23]. However, adapting mobile phone-based programs for use in another context is complex and challenging because of the varied cultural contexts of countries where the intervention is being adapted [24-26]. The adaptation may transcend linguistic translation and extend to cultural themes and values that affect the interpretation and reception of people receiving the messages [24,27].

To ensure that mHealth interventions are effective and reflect both changing technology and users’ needs, it is critical to consider the pathways for adapting mHealth interventions to different populations and settings. This paper describes the processes involved in the adaptation of a successful mobile phone-based smoking cessation program from New Zealand for the Pacific country of Samoa. We reflect on the process of adapting an intervention to a cultural environment with technical and resource limitations. Given the near ubiquitous use of mobile technologies in the Pacific region [28], mobile health (mHealth) and mobile smoking cessation (mCessation) specifically, could be an innovative technology-based alternative to the existing initiatives or occupy a void where there are little options [23]. Previous studies on the use of mHealth interventions in LMICs have identified success in improving the uptake of behavioral change (for example, reducing tobacco use and improving diet) [29]. Yet, the arguments in favor of increasing the technology potential, including apps and wearables, are emerging but with caveats regarding cost and access [30]. Low-resource settings, such as Samoa, may have an impressive mobile penetration rate, but preferences for and levels of mobile use for personal behavior change are not well understood. Furthermore, the issue of equity is important to consider in the context of LMICS—who has access to smartphones is influenced by age, gender, and affordability. We specifically opted to explore the use of text messages as a vehicle for the delivery of smoking cessation support because of the opportunity to engage wide participation and potential scale-up. To this end, we adapted a New Zealand-designed program, which was relatively affordable and potentially modifiable in terms of message content, timing of delivery, and other key cultural distinctions.

Samoa, having reached one of the highest rates of mobile phone coverage in the Pacific region in recent years [28], is well placed to initiate mobile phone-based health interventions. With increased government support, Samoa has made progress toward reducing the percentage of adult smokers from 34.6% in 2002 [31] to 25.6% in 2013 [32,33]. However, as environmental and social risk factors for the early uptake still present a significant challenge [34], mCessation presents an attractive, cost-effective option to deliver health messages. Several factors suggest that mCessation may be a viable motivator to quit smoking in Samoa: the high acceptability of the mCessation program among Pacific populations in New Zealand [30], the empirical evidence of its efficacy [15,35], and increasing mobile phone coverage in Samoa [28].

In this study, we adapted a text-based smoking cessation support program called STOMP (stop smoking with mobile phones) for pilot-testing in Samoa. STOMP was designed in New Zealand to deliver targeted, theoretically sound SMS text messages to people who wanted to quit smoking; this original program demonstrated success in a randomized controlled trial by doubling quit rates after 6 weeks compared with unassisted quit attempts, that is 239 (28%) versus 109 (13%), respectively, (*P*<.001) [15].

STOMP has been adapted and used in the United Kingdom and Argentina [35,36]. In the United Kingdom, the adaptation process of STOMP included reviews by smoking cessation counselors and focus group among potential participants (ie, smokers, ex-smokers, and smokers trying to quit) [37]. In a large randomized controlled trial, the adapted and modified SMS text messaging program (txt2stop) doubled biochemically verified continuous abstinence in 6 months compared with unassisted quit attempts, that is, 268/2911 (9%) versus 124/2911 (4%), respectively, (*P*<.001) [16]. The adaptation in Buenos Aires, which included translation and back-translation, expert review, and discussions among potential users, emphasized that nuances in tone and translations may negatively affect the acceptability. Hence, conducting a formal cross-cultural adaptation is a necessary step in the adoption of smoking cessation interventions [36].

The original STOMP design was based on effective brief intervention methods and social cognitive theory, particularly around enhancing self-efficacy for quitting [15] and was retrospectively referenced against the Michie’s Behaviour Change Technique Taxonomy [38]. The suite of messages was tailored to recognize the iterative phases that are typical, but not universal, in the process of behavior change: contemplation (prep for quit date), action (quit day), and maintenance (relapse support) [39].

In Samoa, this project aimed to adapt and pilot a text-based smoking cessation program called TXTTaofiTapaa (TxtStopSmoke); the process involved identifying the nuances within Samoan cultural context and values important for adaptation of the tool with the overarching intention of increasing smoking cessation attempts among participants. This paper aimed to present insights and lessons learned from the adaptation and implementation of the TxtStopSmoke program.

## Methods

### Adaptation Design

The adaptation process included the following three major steps: (1) preliminary translation and adaptation design; (2) adaptation test and formative evaluation by users; and (3) linguistic and cultural adaptation and refinement. The design process of the adaptation was dynamic, taking into consideration the user experience as an essential part of the adaptation outcome. As such, the adaptation was responsive to the sociocultural context of Samoa and Samoan smokers. Researchers from the University of Auckland collaborated closely with the Samoa Ministry of Health and the National University of Samoa to provide feedback and finalize the translation to the local language. Ethical approval for this study was granted by the University of Auckland Ethics Committee and the Samoa Ministry of Health Research Committee (reference 016631).

### Step 1: Preliminary Translation and Adaptation Design

Initially, 14 SMS text messages from the STOMP program were selected for pretesting. SMS text messages were extracted from each of the STOMP mCessation program phases, based on the Theory of Behavior Change model [39]; these included messages based on the countdown to quit, quit day, intensive phase, and relapse prevention phase [15]. The messages included a combination of motivational messages to persevere with the quit attempt, reminders of the benefits of quitting smoking, and provided information about the consequences of smoking, how to quit and stay quit. These messages were translated verbatim to Samoan. Following the translation, SMS text messages that exceeded the 160-character limit were paraphrased, but the overall content and tone were retained.

### Step 2: Preliminary Adaptation Test and Formative Evaluation by Potential Users

The recruitment of focus group participants was conducted in the capital of Samoa, Apia. Participants, including smokers and ex-smokers, were recruited through posters placed in high people-traffic areas. The participant inclusion criteria were as follows: being a smoker or an ex-smoker (defined as having quit in the last ≥6 months), being aged ≥16 years, having access to a mobile phone, being able to receive SMS text messages for 1 week, being able to provide written informed consent, and participating in two 1-hour group interviews (before and after receiving the SMS text messages). Although >40 people expressed interest in participating, several were excluded because of the lack of mobile phone access or because they did not arrive at the focus group venue. Others were unable to be available to receive the messages for the 1 week required for participation. Notably, 36 people directly contacted the research assistant to participate in the study and fulfilled the eligibility to participate. Participants were grouped according to the demographic homogeneity (age and gender) [40] and smoking status (Table 1). This strategy was used in recognition of traditional Samoan-speaking customs [41] and to leverage on participants shared experiences.

All 6 focus groups were moderated by a Samoan researcher (HT). Participants completed a brief demographic and tobacco use questionnaire at the beginning of each focus group. During the focus groups, the following topics were included in the discussion: beliefs about tobacco use, mobile phone use and preferences, including phone sharing, typical daily use, perceived benefits, and problems with mobile for messages to support quitting. All participants received 20 tala (approximately US $8) following their participation in the interviews.

Following the in-depth discussion on the participants’ views of tobacco use, smoking initiation, and quitting strategies awareness [42], 36 participants agreed to received SMS text messages for 1 week (2 SMS text messages per day) on their mobile phones. A sample of 14 SMS text messages was sent to participants by the research assistant on a prepaid mobile phone. This process aimed to provide qualitative information on the potential users’ attitude toward the mobile phone use and explore their views on the mCessation program within their local context.

After 1 week, participants reconvened for a follow-up group discussion to provide feedback. The follow-up interviews aimed to draw participants’ general feedback on the SMS text messages they received, with particular attention to the content, tone, comprehension, and acceptability. The follow-up interviews covered the following topics: preferences for the program, technical issues, phone and message sharing, preferences for the timing of messages, message engagement, and suggestions for improvements in the messages and the timing of delivery.

The focus groups were digitally recorded, transcribed, and subsequently translated into English for non-Samoan researchers. The results were analyzed using a general inductive thematic analysis method [43]. Two researchers (HT and EU) independently performed an initial coding, which was later categorized and organized into themes by the research team.

### Step 3: Linguistic and Cultural Adaptation

An initial linguistic translation process required all messages to be translated to create a full suite of messages. The translation process was iterative, drawing upon both the content of interviews and consultation with participants regarding the message content and tone. Rigorous consultations with stakeholders were undertaken to ensure the appropriateness and suitability of messages to local setting or preferences.

Messages were delivered at various transition and maintenance stages of the program—countdown to quit day, quit day, 4-week intensive phase, and relapse prevention. Table 2 presents examples of messages created for each of the 4 stages of behavioral change. The SMS text messages were available in both English and Samoan and were then developed into a single, predefined quit day for the pilot.

**Table 1:** Demographics, smoking status, and mobile phone use (N=36).

Characteristics			n (%)
**Demographics**			
	**Gender**		
		Male	22 (61)
		Female	14 (22)
	**Age (years)**		
		16-25	19 (53)
		>25	17 (47)
**Smoking status**			
	Smoker		27 (75)
	Ex-smoker		9 (25)
**Mobile phone use**			
	**Sharing a mobile phone**		
		Yes	14 (39)
		No	22 (61)
	**Smartphone ownership**		
		Yes	19 (53)
		No	11 (31)
		No answer	6 (17)
	**SMS^a^ text message use**		
		Everyday	21 (58)
		Most days	11 (4)
		Few times a week	6 (17)
		Few times a month or less	1 (3)
		No answer	1 (3)

^a^SMS: short message service.

**Table 2:** Example of short message service text messages translation and adaptation for each stage of the program.

Stage in mCessation^a^	English message example	Samoan message example
Countdown to quit day—2 messages per day for 1 week. 15 messages in total including welcome messages.	Talofa lava! Welcome to the TXTTaofiTapaa programme. We will send you texts to help you stop smoking. Seven days to go until you quit!	Talofa lava! O le polokalame lenei o le “TXTTaofiTapaa” o le a lafo atu ni feau tusitusia e fesoasoani i le tuu o lou ulaula tapa'a. Toe 7 aso tuu loa le tapaa!
Quit day—3 messages.	TXTTaofiTapaa: This is it, (name)! QUIT DAY. Mark it! Throw away all your smokes, today is the start of a new beginning. Be strong.	TXTTaofiTapaa: (Name), o le aso lenei e TUU AI LOA LOU ULAULA TAPAA. Tiai loa lau pepa sikareti o se amataga fou lenei moo e. Faamalosi.
4-week program.	TXTTaofiTapaa: Your withdrawal symptoms are probably high right now. Avoid old smoking places.	TXTTaofiTapaa: Masalo ua faateleina lagona o le fia ula i se tapaa. Faaauau pea le tuu o le ulaula tapa'a. Alo ese mai nofoaga e masani ona e ulaula ai.
Relapse prevention—6 weeks of 3 messages per week. 18 messages in total.	TXTTaofiTapaa: (Name), some old smoking situations will keep coming up again and again. You know how to deal with cravings & temptations.	TXTTaofiTapaa: O lagona o le fia ula i se tapa'a e tupu i nisi taimi. Ua e iloa le gaioiga e fai pe a lagona le fia ula i se tapaa. Tetee i le tapaa.

^a^mCessation: mobile smoking cessation.

## Results

### Themes

The themes that informed the development of the adaptation design were described accordingly: (1) initial program perception; (2) message tone and content; (3) message delivery; and (4) perception of the value of mCessation program in general. The results below reflect participants’ perceptions of the messages after receiving them for a week.

### Initial Program Perception

Before receiving an actual program of SMS text messages, participants were shown examples of 3 SMS text messages that they were to receive and then asked about their thoughts regarding the concept of a SMS text messaging program to help them quit smoking. Overall, participants were generally positive toward the idea of receiving supportive messages and concurred the program would offer support to Samoans who smoke.

Good thing to try and see how and would it work. To inform me of the harms that smoking can do and will see how it works. Reading just one of these messages just makes me think about my smoking.

Female smoker, aged >25 years

However, a few expressed doubts about the SMS text messages’ effects, comparing them with antismoking advertisements previously viewed on television or billboards.

Some of those billboards about smoking don’t affect me at all. They shouldn’t follow overseas things, because they just cut and paste it and add a Samoan face but they should do their own.

Female smoker, aged >25 years

These participants suggested that messages should be personalized and tailored to Samoans for greater impact on users.

### Message Tone and Content

During the follow-up focus group discussions, many participants preferred a positive tone of the SMS text messages—encouraging messages that reflected the physiological challenges when quitting were perceived to be helpful. Practical suggestions on what to do to avoid smoking were appreciated (eg, “Get lots of rest to keep your energy up to fight the cravings. If you get tired, it’s easy to have cravings for a smoke.”), as well as those that highlight the benefits of quitting smoking to their family.

The texts that are most helpful were the ones that showed me how to do things…like don’t be around people who smoke. Practical advices were helpful.

Female smoker, aged >25 years

I can remember also the one where if you stop smoking, you have more time with you family, and it saves money.

Female smoker, aged >16 years

Some participants preferred SMS text messages that contained information about the negative effects of smoking—the shock effect perceived to be a necessary motivator for attracting attention and potentially nudging behavioral change. Some requested photo evidence to be included with the SMS text message. Notably, these suggestions were more likely to come from older males in groups. Furthermore, visual representations of the message were considered important, partly for validation; seeing is believing.

But for me, our country if it comes like that [funny], then they won’t take it seriously. What is important is to scare people and [to say] that it will affect them so that they take it seriously. Even the young ones that have just started smoking, if they get this message, then they’ll know how important it is to stop smoking.

Male smoker, aged >25 years

Some SMS text messages stood out among participants and elicited discussion. For instance, many participants disagreed with the SMS text message that encouraged going to public places to avoid smoking, saying that public places are where smokers congregate and many start smoking.

The text about going to public places where there are no smoking, well, there are laws now to not smoke in public places, but it is still going on, like smoking on buses and so on. So, that text might not be appropriate.

Male smoker, aged >16 years

Another message aroused humor and curiosity; that cigarettes contain chemicals found in toilet cleaner. Although some questioned the validity of that specific message, others suggested that these were the most impactful—clear linkages between smoking, the chemicals, and the risk to body organs.

There was one that I got, and I read it to my family, and they thought it was funny and didn’t believe it. The one about the chemicals that are in smoking are also used to clean toilets, and they didn’t believe me. And they said they’re all lies.

Ex-smoker, aged >16 years

Participants preferred SMS text messages that did not use abbreviated slang language, with some commenting that these SMS text messages will be difficult to understand among older smokers. Furthermore, some participants suggested providing the option for smokers to choose between receiving English and Samoan SMS text messages.

### Message Delivery

The timing of message delivery was also carefully considered within the groups. When asked about the time of the day they prefer to receive the SMS text message, participants broadly agreed that sending messages at midday (after a meal, which is a trigger to smoke) and in the evening (when family is around and after dinner) were the most beneficial times.

Time that is appropriate is times of meal times, like the morning, lunch and dinner. Then I can read and change my mind about smoking.

Male smoker, aged >16 years

It’s good at the beginning in the morning and then at the end of the day when people are unwinding, and people are wanting a break and craving a smoke.

Female smoker, aged >25 years

Generally, participants suggested avoiding times when the messages would be disruptive to important events (Bingo night, church services) or rituals (during a meal). Most participants reported being satisfied with the timing of messages and the frequency. Some even suggested they would like to receive more messages to assist them to quit smoking.

### Perception of the Value of Short Message Service Text Messages

After 1 week of receiving messages, many participants reported that they had reduced the number of cigarettes they smoked. A few said nothing changed, but expressed that the SMS text messages increased their intention to quit.

A lot of use of the program for me especially for my family and my relationship with my wife. Because it has given me good thoughts and saving money…I feel better now…I have reduced smoking because it is cheaper, and better for my family. It is important for all Samoans to stop smoking.

Male smoker, aged >25 years

The majority of participants who received the messages were positive about the potential of the program in assisting them to quit smoking. Many felt that, in the absence of any alternative tobacco cessation support (eg, nicotine replacement therapy), messages were likely to be helpful in prompting thoughts about quitting.

Samoans take a while to change because of the way they feel, and it will take a long time to change, but the program is there and helps straight away, and so the program is quicker in helping quit attempts, when they get text messages.

Male smoker, aged >16 years

Phone sharing in Samoa and the close connectivity with family was believed to help in spreading the message. A number of participants reported that they shared the SMS text messages with family or friends, through either actual forwarding the SMS text message or relaying the content of the SMS text messages to family and friends.

I think that this programme is good and so if it is good for me, then it is good for my family and friends. I shared it with others.

Female smoker, aged >25 years

## Discussion

### Principal Findings

The cultural translation and adaptation of the STOMP program was a necessary first step to implementing an mCessation program in Samoa. The value of undertaking a cultural translation cannot be underestimated, with strong evidence of its marked contribution to the acceptability and effectiveness of mHealth interventions [24,44,45]. The current literature provides some guidance on how to adapt mobile phone-based interventions (for use in countries wherein it was not specifically designed), acknowledging the essential role of the end user in this process [24,46,47].

A sample of Samoan smokers and ex-smokers were involved in the process of cultural translation and adaptation of a mCessation tool designed for use in Samoa. Smokers were essential as key informants on design-related decisions such as which messages to include or revise and the timing of message and themes highlighted in the SMS text messages (eg, benefits to the family, negative effects of smoking, practical suggestions to avoid smoking). Previous qualitative analyses affirm the critical role of the social environment, familial connections, including smokers, and perceived barriers and motivations for quitting; this led to minor revisions of the original STOMP messages to include more reference to family (eg, messages about the benefits of quitting smoking to family), and changes to suggested activities and snacks (reduce craving to smoke). Participants identified when cravings to smoke were the highest and when SMS text messages were most useful in providing timely encouragement. The positive response of participants to the program, including the increased focus on quitting or reducing the consumption of tobacco products, was consistent and provided modest evidence that Samoans smokers are likely to use the mCessation program if the program is scaled up and made available as an option for smoking cessation.

One challenge during the initial translation was to retain the integrity of the message while ensuring that the messages were kept within 160 characters. As Samoan words are typically lengthier than their English equivalent, alternative words were selected.

The involvement of key stakeholders and potential implementers of the mCessation program was critical for the formative linguistic translation and subsequent refinement of the SMS text messages. Colantonio et al [36] conducted similar consultations, mostly with tobacco cessation experts, to validate their translated messages for suitability and relevance to the local setting. The process of translation required considerable time and effort, a step that could potentially be underestimated. Our consultations with experts improved the suitability of messages to the local setting, as well as the accuracy and integrity of translation and language use. The translation quality was determined according to the integrity of the translator, with the primary challenge being the identification of the definitive authority in translation [48]. In our experience, New Zealand-based Samoan translators were the least preferred over local officially appointed translators.

### Strengths and Limitations

An important aspect of our cultural adaptation process and one of the strengths of this study is integrating both a “bottom-up” and a “top-down” approach; this process underscores the vital role of the end user of mHealth tools, in this case smokers, to provide inputs about the shape and design of the adapted program. It also recognizes the involvement of key stakeholders who can determine the local acceptability and appropriateness of the intervention. Another strength of this study is partnership with the Ministry of Health Samoa to undertake the process of adaptation and the fact that this work reflects the reality of adapting an SMS text message program for a limited-resource environment.

A local coinvestigator, fluent in Samoan, conducted the interviews. Although this adds strength to the integrity of the data collection, it might have introduced a social desirability response, owing to the tendency to offer affirmative opinions when researching within socially connected group settings.

The aim of this study reflects an appreciation that culturally adapted messages will increase engagement, a predictor of the positive impact of SMS text message programs on health [45,49]. Similarly, engagement with SMS text message interventions is recognized as important to uptake and behavioral change [27]. Behavioral change is a complex process, which despite efforts to define a predictable, universal model remains largely elusive. However, what our work has shown is that careful attention to the nuances of the content and delivery timing of SMS text messages for behavioral change is important.

### Conclusions

The current provision under the Samoa Tobacco Control Act (2013) covers smoke-free environments, tobacco advertising, and plain packaging [50]. However, most tobacco control initiatives in Samoa have been focused primarily on regulating the sale and distribution of tobacco products and tobacco marketing, with some enforcement at the local level [51]. New Zealand, under its bilateral agreement with Samoa, offers support to Samoa to achieve its health goals and targets [52]. Within this scope, we worked with the Samoa Ministry of Health to adapt a mobile phone-based smoking cessation intervention to support Samoan smokers to quit. Lessons learned and observations made throughout the process of adapting the intervention provide value for others attempting similar efforts. Adapting mobile phone-based interventions requires considerable time and resource and keen attention to the nuances of language and meaning—as well as partnerships. Hence, this study suggests that this investment is essential for meaningful improvements to the integrity of technology-based health interventions.

## Abbreviations

LMICs: low- and middle-income countries
mCessation: mobile smoking cessation
mHealth: mobile health
SMS: short message service
STOMP: stop smoking with mobile phones
